# “Chiropractic is manual therapy, not talk therapy”: a qualitative analysis exploring perceived barriers to remote consultations by chiropractors

**DOI:** 10.1186/s12998-021-00404-2

**Published:** 2021-11-25

**Authors:** Shane Derbyshire, Jonathan Field, Jane Vennik, Marc Sanders, Dave Newell

**Affiliations:** 1 Private Practice, Norwich, UK; 2 Centre for Primary Care and Population Studies, University of Southampton, Southampton, UK; 3 AECC University College, Bournemouth, UK

**Keywords:** Remote consultation, Chiropractic, Survey, COVID-19, Coronavirus, Telehealth, Telemedicine, Musculoskeletal, Qualitative analysis

## Abstract

**Background:**

Remote consultations (RCs) enable clinicians to continue to support patients when face-to-face appointments are not possible. Restrictions to face-to-face care during the COVID-19 pandemic has accelerated a pre-existing trend for their adoption. This is true for many health professionals including some chiropractors. Whilst most chiropractors in the UK have used RCs in some form during the pandemic, others have not. This study seeks to understand the views of chiropractors not using RCs and to explore perceived potential barriers.

**Methods:**

A national online survey was completed by 534 registered practicing UK chiropractors on the use of RCs. Respondents had the opportunity of providing open-ended responses concerning lack of engagement in RCs during the COVID-19 pandemic. Textual responses obtained from 137 respondents were coded and analysed using thematic analysis.

**Results:**

The use of RCs provided an opportunity for chiropractors to deliver ongoing care during the COVID-19 pandemic. However, many chiropractors expressed concern that RCs misaligned with their strong professional identity of providing ‘hands-on’ care. Some chiropractors also perceived that patients expected physical interventions during chiropractic care and thus considered a lack of demand when direct contact is not possible. In the absence of a physical examination, some chiropractors had concerns about potential misdiagnosis, and perceived lack of diagnostic information with which to guide treatment. Clinic closures and change in working environment led to practical difficulties of providing remote care for a few chiropractors.

**Conclusions:**

The COVID-19 pandemic may have accelerated changes in the way healthcare is provided with RCs becoming more commonplace in primary healthcare provision. This paper highlights perceived barriers which may lead to reduced utilisation of RCs by chiropractors, some of which appear fundamental to their perceived identity, whilst others are likely amenable to change with training and experience.

## Background

Telehealth is the use of electronic and telecommunication technologies for providing patient care. One form of telehealth, virtual or remote consultations (RCs), allows healthcare providers to communicate with patients remotely, via voice and/or video. These digital approaches in healthcare have experienced a slow but steady growth over the past two decades [1–4]. They are felt to bring potential benefits in access to healthcare, reduced travel time, reduced non-attendance, increased patients’ self-awareness, ability to self-manage and lower costs [5–10]. Problems reported with RCs include technical issues, perceptions around clinically inadvisability, provision of impersonal care, and potentially creating barriers to the development of a good clinician-patient relationship [7, 10, 11].

The start of the global COVID-19 pandemic in 2020 posed a challenge to provision of healthcare due to the way in which the SARS-CoV-2 virus is transmitted, especially for clinicians who provide manual therapy which is close-contact by nature. SARS-CoV-2 virus particles are spread more easily in indoor settings due to poorer ventilation, close range distances of less than 1 m, and through direct contact [12] all of which are common features of the clinical setting and interventions that manual therapy clinicians work within and provide. In response to the pandemic and in line with government guidelines, some UK national chiropractic organisations issued statements recommending the pause of face-to-face consultations in March 2020 [13–15]. The self-evident benefits of RCs in the presence of a viral pandemic such as COVID-19 is that they remove the risk of transmission of infection and allow for clinical encounters when patient or care giver are unable to travel to attend a clinical setting.

Like those from other countries, some UK chiropractors turned to RCs to continue to support their patients when face-to-face care was no longer an option [16]. The use of RCs pre, during, and post the COVID-19 pandemic and the views of chiropractors towards RCs were explored in a survey distributed to all registered UK practicing chiropractors [17]. The cross-sectional survey, developed and administered by MS, JF, and DN was designed to evaluate the frequency and pattern of use of RCs by the UK chiropractic profession during the pandemic, and to explore attitudes to their wider use in chiropractic healthcare provision. Whilst two thirds of the 534 survey respondents reported adopting or planning to adopt a form of RC during the pandemic, a third had no plans to implement RCs in their practice.

The aim of this nested qualitative work was to explore the perceptions, views and perspectives of chiropractors who had not adopted RCs as a method of consulting during the pandemic, and to understand potential perceived barriers to their wider implementation in UK chiropractic practice following the COVID-19 pandemic.

## Methods

An online national cross-sectional survey was sent to all registered practicing chiropractors in the UK in May 2020 (n = 3131). Requests to complete the survey were sent via email by the UK national registration body, the General Chiropractic Council (GCC) as well as national member associations (British Chiropractic Association (BCA), McTimoney Chiropractic Association (MCA), United Chiropractic Association (UCA), and Scottish Chiropractic Association (SCA)). Potential participants were provided with information about the study prior to recruitment and were informed that submitting the completed survey would constitute consent to participate. This report is one of two reports derived from this cross-sectional survey, the other report [17] addresses the quantitative aspects of the survey, further details as to the design and dissemination of the survey can be found in this report. A total of 534 chiropractors completed the survey and 137 provided textual responses to open ended questions regarding the use of RCs. In this paper, we report on the qualitative analysis of the free-text responses to the survey question about barriers to RCs during the COVID-19 pandemic.

### Data collection and analysis

Survey respondents who reported not providing RCs were asked to provide details using free-text responses. Survey responses were extracted from the survey data into Microsoft Excel.

Analysis commenced through familiarisation with the dataset. The textual responses were read, organised and coded using open-label coding independently by SD and JF—both practicing chiropractors and researchers—in Microsoft Excel. These were consolidated to a common code set through discussion by these two authors. Codes were then mapped together, and overarching themes emerged through further discussion with JV, a qualitative research fellow and DN, a professor of integrated musculoskeletal (MSK) care, bringing multiple perspectives to the analysis. A pragmatic approach was taken in this study.

### Ethics

This study received approval from AECC University College Ethics Sub Committee on the 13/05/2020 (#E124/05/2020).

## Results

### Participants

Of the 534 responses to the survey 175 (32.8%) self-identified as not using RCs and 137 provided textual information as to why this was. Free-text responses varied in length from 3 to 467 words (mean 33 words), with the majority providing sufficient detail to explain their reasons for not providing RCs. Respondent characteristics are described in Table 1. The majority were female (58%), aged 30–59 and have been in practice for more than 11 years. The characteristics of participants in this sample were similar in terms of age range, gender, chiropractic association membership and years in practice to those in the full survey dataset.

**Table 1:** Description of respondent characteristics providing textual data in the survey compared with total survey participant characteristics

Variable	Study participants (137)		Survey participants (534)	
	n	Percentage	N	Percentage
Age				
21–29	18	13.1	62	11.6
30–39	29	21.2	147	27.5
40–49	47	34.3	146	27.3
50–59	29	21.2	128	24.0
60–69	13	9.5	43	8.1
70–79	1	0.7	7	1.3
Gender				
Female	79	57.7	280	52.4
Association				
BCA BCA	72	52.6	330	61.8
UCA	24	17.5	68	12.7
MCA	13	9.5	64	12.0
SCA	12	8.8	40	7.5
Other	16	11.7	31	5.8
Years in practice				
0–1 years	3	2.2	16	3.0
2–5 years	22	16.1	71	13.3
6–10 years	18	13.1	100	18.7
11–15 years	24	17.5	87	16.3
16–20 years	30	21.9	111	20.8
21–30 years	27	19.7	96	18.0
31–40 years	12	8.8	43	8.1
41–50 years	1	0.7	8	1.5

### Themes

**Fig. 1:**
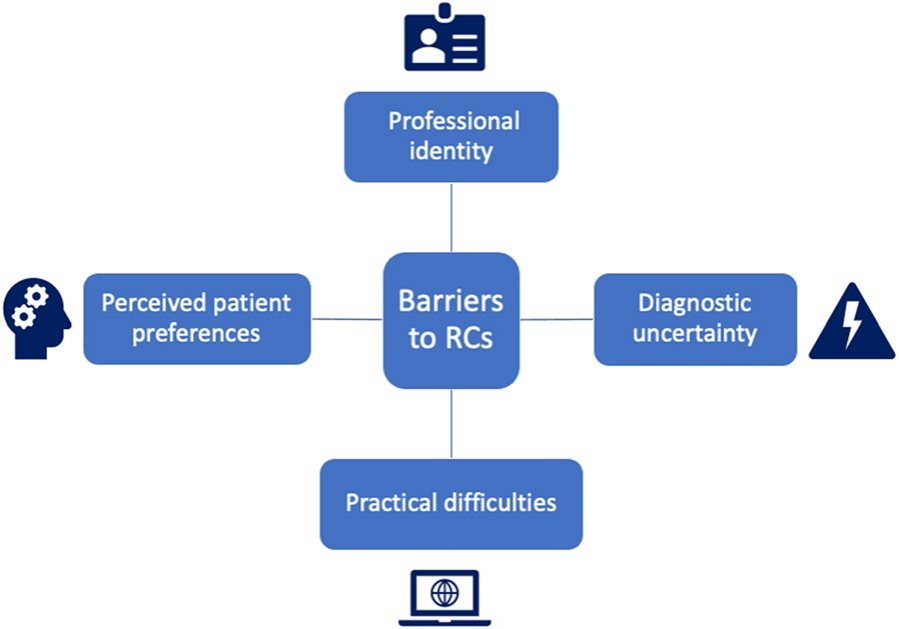
Conceptual model of perceived barriers to utilisation of remote chiropractic consultations

We identified 4 themes in our analysis: (i) professional identity, (ii) diagnostic uncertainty, (iii) perceived patient preferences, and (iv) practical difficulties. Each theme is described in detail below and quotations are used as exemplars with an identifier for respondent number and member association (e.g., R1—SCA). Non-standard capitalisations for words such as “Chiropractic” or “Chiropractors” were present in respondent text. Figure 1 gives a diagrammatic representation of these identified themes.

### Professional identity

Many respondents reported that provision of RCs did not align with their strong sense of identity as providers of physical hands-on care. Some commented that the essence of chiropractic is encapsulated within the roots of its name: "Chiropractic comes from the Greek done by hand" (R14—SCA). Some participants stated, in general terms, that RCs present a barrier to providing this style of care: "My work is hands on that cannot be done remotely" (R34—SCA). Others pointed to specific treatments that they perceived as essential to chiropractic care yet self-evidently impossible to provide in RCs, such as spinal manipulation to correct subluxations. "You can't talk a subluxation away, neither can you provide Chiropractic (by hand) care over the phone" (R19—BCA).

Some respondents explicitly juxtaposed chiropractic with other healthcare professions that they portrayed as more amenable to incorporating RCs. Some, for example, mentioned that RCs may be more appropriate for psychotherapy: "Chiropractic is manual therapy not talk therapy… If someone needs CBT this should be done by a CBT practitioner" (R4—UCA). A few participants explicitly contrasted their work, perceived as inescapably manual, with that of physical therapists who could use RCs to provide advice and/or suggest exercises: "Exercises are the remit of physiotherapists" (R136—UCA) "I don't want to be just an advice and exercise giver" (R78—BCA).

### Diagnostic uncertainties

Many respondents expressed concerns about reaching a safe diagnosis during RCs. “I believe you should carry out a thorough case history and physical examination before you make any diagnosis” (R40—UCA). Without the ability to perform an adequate physical examination during a RC, a few respondents were concerned about the possibility of missed diagnosis or red flags. “I fear you would miss important red flag since you are unable to do a physical examination” (R76—UCA).

There was also a concern that an inaccurate diagnosis may result in incorrect advice being given to the patient. “Whilst I may be able to offer basic self help advice, the inability to perform a thorough assessment of my patients condition could result in incorrect and inappropriate advice with potentially catastrophic outcomes” (R131—BCA).

### Perceived patient preferences

Some respondents perceived a lack of patient demand for RCs. A few suggested that uptake in their clinic would be limited if the service was offered. “my patient base is such I would strongly doubt many would want a remote consultation” (R65—BCA).

There was a general perception among many respondents that patients prefer face-to-face consultations and are expecting a hands-on treatment when they have a consultation with a chiropractor. “Patients don’t see a Chiropractor for a chat and advice, they come for a specific Chiropractic adjustment with full focus and intent” (R135—No member association).

A significant number of respondents questioned the ethics of charging for RCs resulting in a disinclination for providing that service. “I do not feel as though I could ethically charge for it, and therefore I don’t want to do it” (R42—BCA).

### Practical difficulties

Practical difficulties in providing RCs was an important reason as to why chiropractors are not providing RCs. Due to restrictions imposed by the UK government in response to the COVID-19 pandemic many chiropractors were working at home which for some meant a lack of space or uninterrupted time. This was expressed in a few of the respondents’ answers where they found it difficult to find a quiet and confidential place to work away from their family “I do not have a suitable space where staying with family during lockdown” (R24—BCA). The technology involved in RCs created barriers for a few respondents, including problems with access to sufficient quality internet bandwidths and phone signals to ensure good patient care over video or phone, and lack of access to patient notes for chiropractors who did not use digital notes. Whilst others reported being uncomfortable with using technology for such purposes “I am not ‘technology minded’” (R55—No member association) and lacked the training and/or knowledge of utilising and delivering care via these contemporary technologies.

## Discussion

The use of RCs provided an opportunity for UK chiropractors to deliver ongoing care during the COVID-19 pandemic, whilst meeting the government and national body guidelines for safe practice [13–15]. Many chiropractors, however, express concern that RCs mis-align with their strong professional identity of providing ‘hands-on’ care. Some chiropractors also perceive that patients expect physical treatment when consulting a chiropractor, and thus predict a lack of demand where direct contact is not possible. In the absence of a physical examination, many chiropractors have concerns about misdiagnosis and lack of diagnostic cues with which to guide treatment. The change in working environment during the COVID-19 pandemic, including clinic closures and working from home, has led to practical difficulties of providing remote care to chiropractic patients.

### Professional identity

Professional identity is formed from a core set of beliefs and values that are unique to a particular group or sub-group of professionals [18]. The identity that is formed from these views can contribute toward both unification or division relating to best practice. Identity arose as a substantial theme from many respondents in this study and parallels a wider contemporary issue in the chiropractic profession over what it is exactly chiropractors do or should provide for their patients [19–22]. A recent review on chiropractic identity [23] found that chiropractic professional identity is “complicated” but confirmed the prior purported three chiropractic identity subgroups, which consist of “two polarised approaches and a centrist or mixed view”. It is thought that the division in identity has arisen as scientific investigation started to challenge historical chiropractic paradigms, and some authors have suggested that such divisions in identity centre around the idea, significance, and practice of science itself [24]. Despite these apparent divisions, it is worth observing that divergent factions related to identity are not isolated to chiropractic and is observed in other professional groups also, such as physiotherapy [25] and counselling [26].

The results of this study show that a substantial proportion of chiropractors hold a professional identity in which ‘hands-on’ therapy sits at the heart of the care they provide for their patients and RCs represent a medium of clinician and patient interaction in which this type of therapy cannot be delivered. The circumstances surrounding the pandemic was a difficult pill to swallow for these chiropractors, where clinician and patient alike cannot engage in their usual care for risk of threat to their health and in some cases their mortality through something as basic as human touch. This led to a situation where the benefits of manual therapy interventions did not outweigh the risks of potential viral transmission, especially for individuals within vulnerable groups with comorbidities. Where urgent MSK care was required, chiropractors conducted risk assessments alongside informed patient consent to justify delivering in-person care for a limited number of patients within these groups [13].

Communication as an intervention is not seen by these chiropractors as being at the core of the chiropractic encounter. These respondents consider talking therapy, exercises and advice as secondary forms of care that supplement rather than replace the physical care provided by chiropractors. However, this perception of the adjunctive nature of non-physical interventions conflicts with the evidence-base for lower back pain (LBP) and neck pain, conditions that constitute the vast majority of presentations chiropractors manage on a day-to-day basis [27]. Comprehensive contemporary reviews suggest advice and reassurance as first line care for simple LBP and exercise and cognitive behavioural therapy (CBT) approaches for more complex cases [28]. A recent study found that statistically significant predictors of reduced back-related disability were; a stronger therapeutic alliance, higher patient satisfaction, reduced patient-perceived treatment credibility, and increased practitioner-rated outcome expectancies. Stronger therapeutic alliance demonstrated the largest effect sizes [29]. Other studies [30] have also shown that therapeutic alliance modulates pain in experimental settings using physical therapy and in encounters where spinal manipulative therapy is provided [31]. Given these components are almost certainly present and active in all chiropractic encounters it may be more accurate to describe what a chiropractor provides to the patient as ‘chiropractic care’ (i.e., a multimodal encounter constituting multiple therapeutic factors) rather than a historical description that sees the active component and cause of positive clinical outcomes as spinal manipulation or manual therapy alone [32]. Therefore, although the beliefs of this group of chiropractors precluded their use of RCs, they may potentially be undervaluing the impact of their skills and expertise that they have developed alongside their manual skills such as communication, exercise, and advice—all of which can be delivered remotely.

A minority within this survey, identified with subluxation theories for the justification of manual therapy interventions, which precluded their use of RCs due to its omission of hands-on care. Despite evidence for the detection and correction of subluxations being unsubstantiated [33], there remains a perception amongst some chiropractors that such historical constructs are legitimate. The viewpoint that sees the primacy of adjustments to remove putative subluxations as an accurate description of chiropractic identity is increasingly seen as a historic construct within much of the profession despite continued presence on some chiropractic websites [34] and such ideas remain strongly associated with the education of chiropractors in a minority of chiropractic programs [35–37]. This study shows that, albeit in a small proportion of chiropractors, identification with subluxation theories is still present and has the capacity to act as an obstacle to the use of more contemporary approaches.

Ascertaining clinical and satisfaction outcomes arising from chiropractors delivering RCs would provide key insights into the veracity of beliefs in the primacy of hands-on care as based in clinical reality or merely a historical legacy of the origins of the profession. Indeed, when asked, chiropractic patients undergoing care via RCs remain highly satisfied [38].

### Diagnostic uncertainty

The chiropractors’ concerns regarding reaching a safe diagnosis mirror those of other healthcare professionals trained within systems that emphasize the role of physical examination skills. I.e., some healthcare professionals report feeling cautious in reaching diagnostic decisions without being able to use particular skills during a consultation [39–41]. However, overall remote outpatient consultations are considered to be safe in general practice and other areas of healthcare [7, 42]. A review of emergency department attendance by patients from general practices adopting a ‘telephone first’ system, found a slight decrease in emergency department attendance (2% per annum) for conditions not deemed as requiring emergency care. Thus RCs allowed for a more appropriate use of resources over time [43].

The concern expressed by respondents of this study is reflected in advice given to general practitioners in the UK where the use of ‘safety netting’ advice is emphasised when performing RCs, with a low threshold suggested for arranging face-to-face assessment in the presence of diagnostic uncertainty [44, 45]. This advice could be applied by chiropractors to help to mitigate the risk of missing serious diagnoses.

### Perceived patient preferences

Our study showed that within a group of participants who did not utilise RCs, chiropractors perceived that there was a lack of patient demand for RCs within their patient base, that patients would not expect to receive advice and explanation alone and non-face-to-face approaches when consulting with a chiropractor to manage their pain, and that the value of RCs was not sufficient to ethically charge patients for this service.

These perceptions are at odds with prior work exploring patients’ beliefs when attending chiropractors. For example, Sigrell [46] reported that patients in Sweden expected to receive an explanation for their symptoms and to be given advice regarding exercise from a chiropractor, and further that chiropractors also expected to provide these as part of their care. In the USA, provision of information by chiropractors has been given as a contributing reason for high levels of patient satisfaction with care when compared to other professions [47].

In a recent survey of patients receiving care via RCs with UK chiropractors, none reported dissatisfaction with their RCs [38]. Of those who had seen a chiropractor before, less than 15% were less satisfied with their RC when compared to their last face to face appointment [38]. In other professions such as general practice and physiotherapy, patients are reportedly as satisfied with telephone or video consultations as they are with face-to-face consultations [6, 11], and a large proportion of patients with chronic MSK pain (43%) report a preference RCs over face-to-face visits [10].

However, only the views of chiropractors choosing not to engage in RCs were included in the presented study. It is possible that this subgroup is in part defined by the emphasis they place on manual therapy, which may in turn influence their belief as to the specific component of care that patients value and expect to receive. This therefore may underpin the emergence of a theme suggesting patients don’t value care that does not include a physical component, and may potentially explain their behaviour and decisions not to offer or effectively promote RCs to patients.

### Practical difficulties

Practical barriers to providing RCs emerged as a minor theme, yet they are important factors to consider. The concerns of chiropractors, who are also parents, regarding working from home, have been echoed by many parents in the UK [48]. Their views highlight the real problems involved with providing childcare and home-schooling during the pandemic whilst schools were closed, while at the same time balancing the demands of their working day within the confines of their home.

The limited or lack of infrastructure of their work setup at home whilst clinics were closed clearly made it difficult for chiropractors, and their problems were predominantly centred around access to resources that they would normally have in their clinics but was not available in their home environment. Implementation of strategies such as migration of paper clinical notes to digital notes that allows encrypted access in the home setting outside of private chiropractic clinics may be useful in case of similar future incidents that require the provision of care in non-face-to-face settings.

For chiropractors who are less tech-savvy, this aspect could be addressed by offering training support and resources for chiropractors in use of RCs with their patients in terms of adaptation of clinical space, processes, and communication including consent, as well as the practical aspects of choosing suitable software and hardware to effectively deliver RCs [49]. Chiropractors should ensure that the software that they use is encrypted, and the software’s privacy policies are compliant with data protection regulations (such as the EU General Data Protection Regulation). For patient notes, it is advisable to record additional details about the RC that would not be intuitively recorded, such as technical difficulties encountered during the consultation, the presence of a chaperone, and the name of the software used to conduct the RC.

Clinicians may want to consider how they might adapt their existing clinical processes to an RC setting; this may include the logistics of appointment booking, pre-RC patient information, methods of patient access to the RC, identity checking, remote physical testing, intervention delivery, remotely provided follow-up resources, and processing of payments.

Other considerations include compliance to health and safety legislations both for the patient’s and the chiropractor’s environment to mitigate, for example, risks of falling during movements or activities, and to consider workstation assessment and ergonomics for employees such as admin or clinical staff [50].

### Strengths and limitations

As far as we are aware, this study is the first to explore chiropractors’ perspectives of RCs and provides an important insight into the key barriers for wider implementation in the chiropractic profession. The study was conducted by a multidisciplinary team of chiropractors and academic researchers, bringing a multidimensional perspective to the analysis and interpretation.

Just under 58% of respondents were female which is slightly higher than the equal gender split of chiropractors registered with the GCC (F = 50.02%). Due to heterogeneity of data, it is not possible to comment further on the generalisability of responses received when compared to the population of UK chiropractors.

This study carried out qualitative analysis of free text survey responses which is a methodology that has previously raised concerns in the literature on its limitations [51, 52]. Free text responses may be unrepresentative due to some participants aversion to writing comments. Those that do comment may also represent those with more extreme views- both positive and negative. However, counter arguments suggest that when this form of data is analysed appropriately it can contribute to the generation of rich insights within the topic of interest [53, 54]. This study also involved the analysis of verbatim responses from a single question in a survey of the chiropractic profession, and only sought the views of those who were not currently providing RCs. It would be valuable to explore the views of those who do use RCs and what facilitates these chiropractic consultations. It was also not possible to explore respondents’ meanings in more depth, and further qualitative work would be helpful to explore this in more detail.

Additionally, respondents were self-selecting in choosing to reply to a study about RCs and so may have been more likely to attract responses from those who held strong views about the subject. Our sample contained a slightly disproportionately high response from females with data that was largely provided by those entering the profession over 10 years ago.

## Conclusions

The COVID-19 pandemic may accelerate already underway changes in the way healthcare is provided going forward, with RCs becoming more commonplace in primary healthcare provision. Barriers have been identified to chiropractors adopting RCs, some of which appear fundamental to their identity whilst others are likely amenable to change with training and experience.

## Data Availability

Access to the data will be made upon request.

## Abbreviations

AECC: Anglo-European College of Chiropractic
BCA: British Chiropractic Association
CBT: Cognitive behavioural therapy
DC: Doctor of chiropractic
DN: David Newell
GCC: General Chiropractic Council
JF: Jonathan Field
JV: Jane Vennik
LBP: Lower Back Pain
MCA: McTimoney Chiropractic Association
MS: Marc Sanders
MSK: Musculoskeletal
RCs: Remote consultations
SCA: Scottish Chiropractic Association
SD: Shane Derbyshire
UCA: United Chiropractic Association
UK: United Kingdom
USA: United States of America
